# The diagnostic accuracy of 332 incisional biopsies in patients with malignant tumors in the musculoskeletal system

**DOI:** 10.1186/s12957-022-02883-w

**Published:** 2023-01-09

**Authors:** Michael Dirks, Nicolas K. Ewerbeck, Tobias M. Ballhause, Sebastian Weiß, Andreas Luebke, Carsten Schlickewei, Karl-Heinz Frosch, Matthias Priemel

**Affiliations:** 1 Department of Trauma and Orthopedic Surgery, University Medical Center Hamburg-Eppendorf, Hamburg, Germany; 2 Department of Pathology, University Medical Center Hamburg-Eppendorf, Hamburg, Germany; 3 Department of Trauma Surgery, Orthopedics and Sports Traumatology, BG Hospital Hamburg, Hamburg, Germany

**Keywords:** Incisional biopsy, Core needle biopsy, Soft tissue sarcoma, Bone tumor, Metastasis, Lymphoma

## Abstract

**Background:**

It is known that specimen collection followed by histopathological workup is the core of evidence-based medical therapy of musculoskeletal tumors. There exist many controversies about how a biopsy should be performed. While some centers recommend minimal invasive biopsy procedures, mostly the core needle biopsy (CNB), others prefer the incisional biopsy.

**Purpose of the study:**

This study aimed to determine the accuracy of incisional biopsy for malignant tumors in the musculoskeletal system. Moreover, advantages and disadvantages to other biopsy methods are discussed.

**Methods:**

This retrospective, single-center study about 844 incisional biopsies (benign and malignant) analysis the diagnostic accuracy of 332 malignant tumors, concerning the final histopathological result. In addition, surgical complications are analyzed to find the best way to plan and treat patients timely and correct. Secondary endpoints are the patients age, the pure operation time, as well as the type of tumor, and the subsequent therapy.

**Results:**

In summary, incisional biopsy corresponded a sensitivity of 100% for malignancy in 844 incisional biopsies and a specificity of 97.6% in 332 malignant tumors, but it features greater operative expense (incision/suture 23.5 min) and the risk of general anesthesia.

**Conclusion:**

The method of biopsy should be tailored to the individual patient and the experience of the center performing the procedure.

## Introduction

**Fig. 1:**
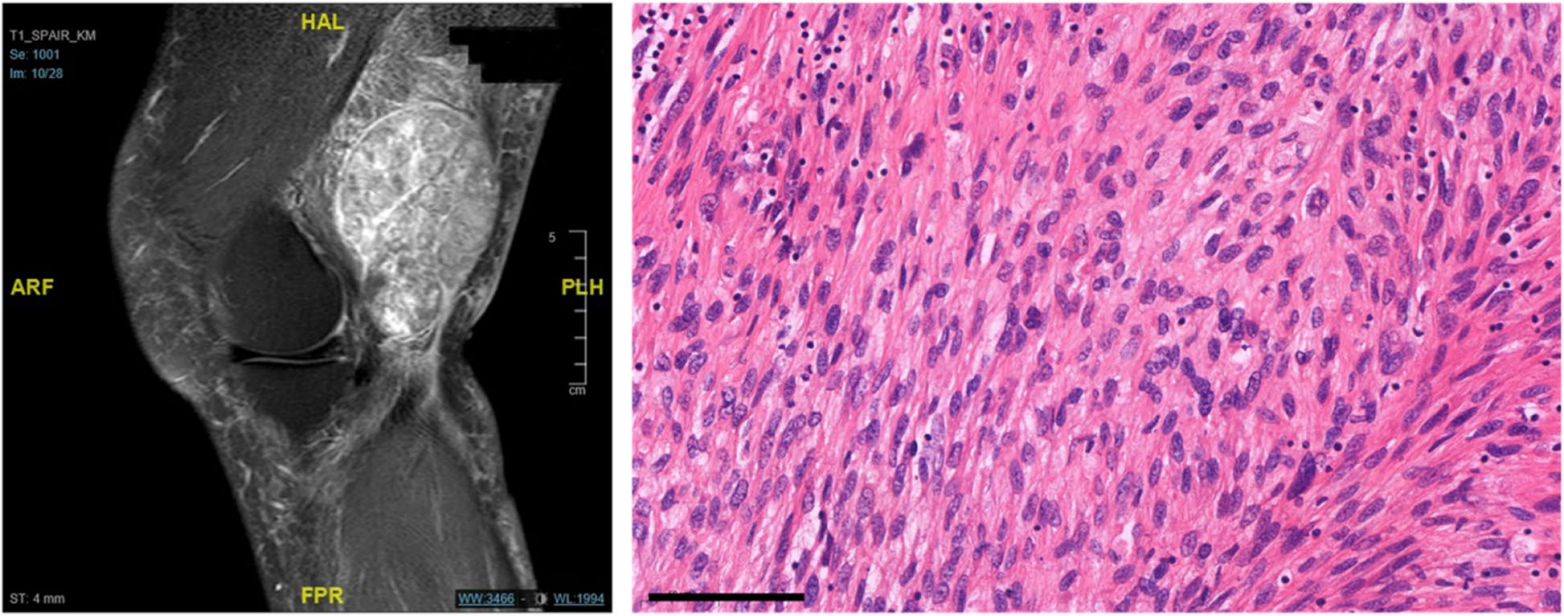
Exemplary images of a high-grade sarcoma. The left image shows the T1-weighted MRI scan with contrast medium in a sagittal plain. An inhomogenous, lobulated tumor can be seen in the left leg of in a 62-year-old patient. The right image shows the high-grade spindle cell sarcoma with features of fibrosarcoma in hematoxylin-eosin stain (bar indicates 100μm)

The biopsy is the critical diagnostic step for the appropriate treatment of all cancerous diseases. It is especially challenging in bone and subfascial soft tissue. Sarcomas are malignant tumors of mesenchymal cells, and they are generally distinguished in soft tissue sarcoma (STS) and bone sarcoma (BS). STS and BS are rare cancers in adults [20]. Because of the rareness of sarcomas and the frequent misinterpretation in imaging, the lesions are mistaken as benign lesions [22]. The most important diagnostic step to identify these heterogeneous tumors is the standardized histopathological preparation after biopsy (Fig. 1). Safe specimen collection is the basic requirement for early initiation of therapy for sarcomas [4]. For this reason, the method of biopsy is always the subject of current discussions. Until now, the guideline on soft tissue tumors generally recommended that a suspicious mass be referred to a center for diagnostic imaging. Furthermore, guidelines advocate open biopsies preferably, but for bone tumors, there are further specifications that need mention. This includes, the incision line being parallel to the line of incision to be taken in the surgery and the scar to be amenable to be encompassed in the final surgical field. If, depending on the possibility, CNB can be performed in a standardized manner in a center, close cooperation between the staff performing the CNB, the pathologist, as well as the subsequent surgeon, and any oncologists providing further treatment is strongly recommended. The importance of proper planning, procedure, and subsequent workup of biopsies of bone or soft tissue has already been described in the literature. Because there is no single standard approach to biopsy for widely varying conditions, biopsy decisions are influenced by personal opinions and judgments. Thus, they are inevitably based on scientific discussion, personal experience, and the expertise of the multidisciplinary team (MDT). A biopsy performed in a center that includes experienced radiologists, pathologists, orthopedic surgeons, and oncologists usually has higher accuracy and a lower complication rate [10]. Furthermore, it might be of great advantage to the patient when the complete treatment of biopsy and consecutive tumor resection lies in the hands of one surgeon. Meanwhile, soft tissue and calcified tissue are not only affected by primary mesenchymal tumors,metastases and tumors of the lymphatic system are frequently diagnosed. Especially, the skeleton is a common site of metastasis. Sex-specific cancer cells (for example, prostate and breast cancer) often settle in the microenvironment of the bone trabeculae [16]. The following osteolysis with accompanying risk of stability can be prevented by systemic therapies, stabilizing surgery and by radiation in curative as well as palliative intention [2, 7]. Finally, due to the hematopoietic function in bone marrow, many primary hematopoietic malignancies can be diagnosed in bone tissue as the initial manifestation [1]. Overall, these four malignant entities must be confirmed by biopsy with subsequent histopathologic workup after a detailed history and imaging diagnosis. The most common and well-established method of biopsy is a surgical incisional biopsy. The guideline for soft tissue tumors puts core needle biopsy (CNB) on an equal footing with surgical biopsy [8]. The Bone Metastases Guideline considers biopsy to be indispensable in cases of the unknown primary tumor, as well as a solitary focus, and finally, the Lymphoma Guideline recommends histopathological examination for individualized treatment of the disease [6]. Regardless of the method of biopsy, it always must yield representative tissue for a complete histopathologic analysis.

The purpose of this retrospective, single-center study is to investigate the diagnostic precision of incisional biopsy for STS, BS, and metastasis as well as lymphoma located in bone or soft tissue.

## Methods

The study had a retrospective, single-center design. The data collection and analysis were anonymous and in accordance with local state law (§17 HmbKG). Institutional Review Board approval was given. All patients were surgically treated from 2011 to 2021 at a university cancer center (UCC). A total of 844 incisional biopsies performed at our institute. To determine the diagnostic accuracy by means of sensitivity and specificity, we decided to evaluate the malignant histopathological results. In these cases, subsequent resection or oncologic system therapy was performed.

Demographic clinical, radiological, and histopathological data were retrieved from the patients’ electronic medical chart. In most cases, radiological imaging was performed by residential radiologists and subsequently, based on the imaging diagnostics, the presentation took place in our musculoskeletal orthopedic center. In some cases, the imaging was performed directly in the UCC.

All incisional biopsies were performed under general anesthesia. After the procedure, patients were observed for at least 2–4 h to ensure the absence of immediate complications, such as dizziness, bleeding, vomiting, or neurovascular injury.

### Inclusion criteria

Adult patients with malignant tumors in the radiologist’s report and the histopathologic report was collected from the electronic medical charts.

### Exclusion criteria

Patients under 18 years of age were excluded in this single-center study, because the UCC treats only adults. Furthermore, all patients with biopsies or incomplete tumor resections, which were performed in other health care institutions and only then referred to the UCC.

All surgical specimen was analyzed in the pathology department of our institution. In cases of doubt, a reference pathology was contacted.

Statistical analysis: The data was analyzed with GraphPad 9 Software (Los Angeles, CA, USA). Parametric distribution was tested by the Smirnov-Kolmogorov test. Different cohorts were compared to with ANOVA. A confidence interval of 95% was chosen. Mean values are followed by standard deviation in (±).

## Results

**Fig. 2:**
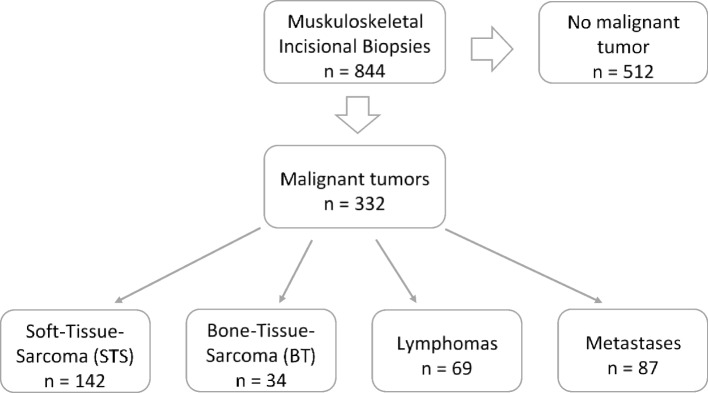
The flow-chart shows the inclusion process of patients for retrospective listing in the study

**Fig. 3:**
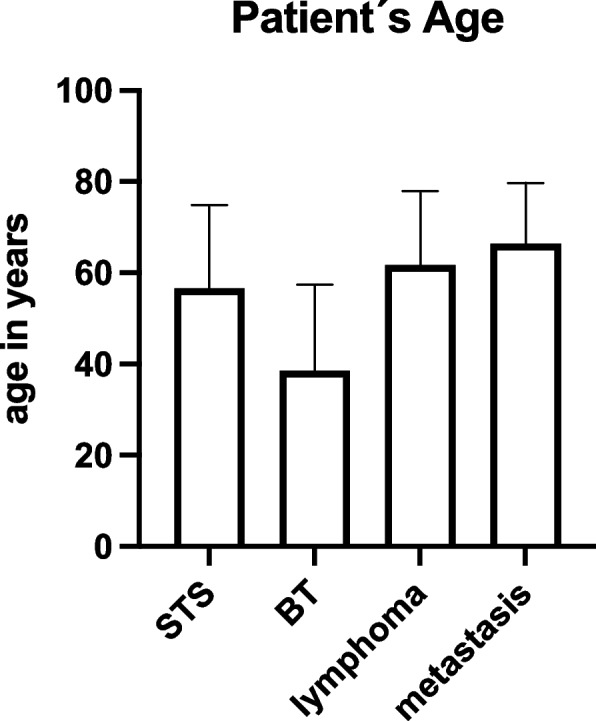
Average patients’ age in relation to about the tumor types

**Fig. 4:**
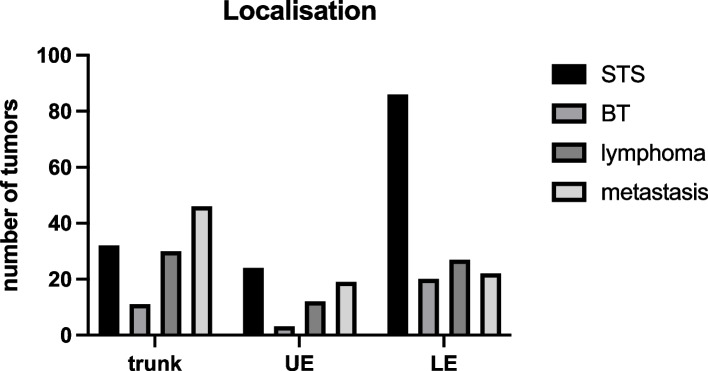
Location of the various tumor types throughout the body

All included patients presented to the UCC were based on imaging suspicion of a malignant mass in soft or bone tissue. After reviewing the previous findings, taking a detailed medical history, examining the patient clinically and, if necessary, performing additional diagnostic tests, we decided to perform a diagnostic surgical incisional biopsy. The mean time between the initial patient presentation and incisional biopsy was 8.8 days (SD: 8.4). 332 individual patients were included to our study, 129 (39.2%) were female and 203 (60.8%) male patients. In 332 biopsies performed, histopathologic workup diagnosed 142 STSs, 34 BTs, 69 lymphomas, and 87 metastases (Fig. 2). STS represent the largest proportion at 42.8%, BT the rarest at 10.2%. Lymphomas were diagnosed in 20.8% of cases, and metastases finally in 26.8%. The 142 patients diagnosed with soft tissue sarcoma were on average 56.6 (±18.3) years old (Fig. 3). In contrast, the 34 patients with bone sarcoma had a mean age of 38.5 (±18.9) years. In the case of diagnosed lymphoma, the mean age of the patients was 61.7 (±16.2) years. Finally, in the case of a proven metastasis, the mean age was 66.5 (±13.2) years. We divided the individual tumor types into three body units, the trunk, the upper, and the lower extremities: This subdivision of tumor type according to sites is presented in Fig. 4. One hundred seventeen (35.2%) biopsies were performed in the trunk. Here, we could diagnose 32 soft tissue sarcomas, 11 bone sarcomas, 30 lymphomas, and 46 metastases. Thus, of the 332 incisional biopsies performed, 9.6% were soft tissue sarcomas in the trunk, 3.3% were bone sarcomas in the trunk, 9% were lymphomas in the trunk, and 13.9% were metastases in the trunk. In addition, 58 (17.5%) biopsies were performed on the upper extremities. Here we could diagnose 24 soft tissue sarcomas, 3 bone sarcomas, 12 lymphomas, and 19 metastases. Thus, of the 332 incisional biopsies performed, 7.2% were soft tissue sarcomas of the upper extremities, 0.9% were bone sarcomas of the upper extremities, and 3.6% were lymphomas of the upper extremities, and 5.7% were metastases of the upper extremities. Finally, most diagnostic incisional biopsies were performed on the lower extremity. These amounted to 155 (46.7%). Here, we were able to diagnose 86 soft tissue sarcomas, 20 bone sarcomas, 27 lymphomas, and 22 metastases. Thus, of the 332 incisional biopsies performed, 25.9% were soft tissue sarcomas of the lower extremities, 6.0% were bone sarcomas of the lower extremities, and 8.1% were lymphomas of the lower extremities, and 6.6% were metastases of the lower extremities.

**Fig. 5:**
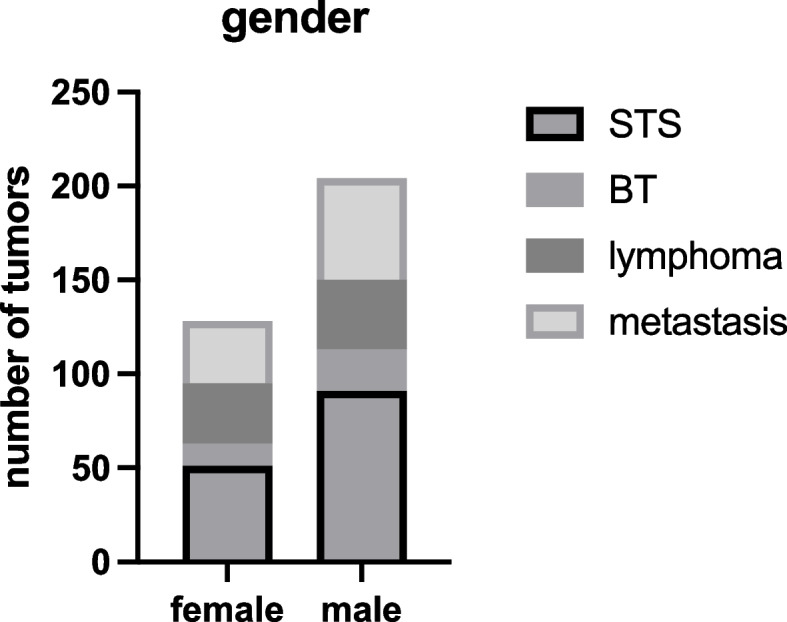
Number of tumors differentiated by male and female patients

In addition, a total of 129 incisional biopsies performed on female patients showed 51 soft tissue sarcomas, 12 bone sarcomas, 32 lymphomas, and 33 metastases (Fig. 5). This means 39.5% of the incisional biopsies performed on female patients were soft tissue sarcomas, 9.3% were bone sarcomas, 24.8% were lymphomas, and 25.6% were metastases. In contrast, we detected 91 soft tissue sarcomas, 22 bone sarcomas, 37 lymphomas, and 54 metastases in the 203 male patients. This means 70.5% of the incisional biopsies performed on male patients were soft tissue sarcomas, 17.1% bone sarcomas, 28.7% lymphomas, and 41.9% metastases.

**Fig. 6:**
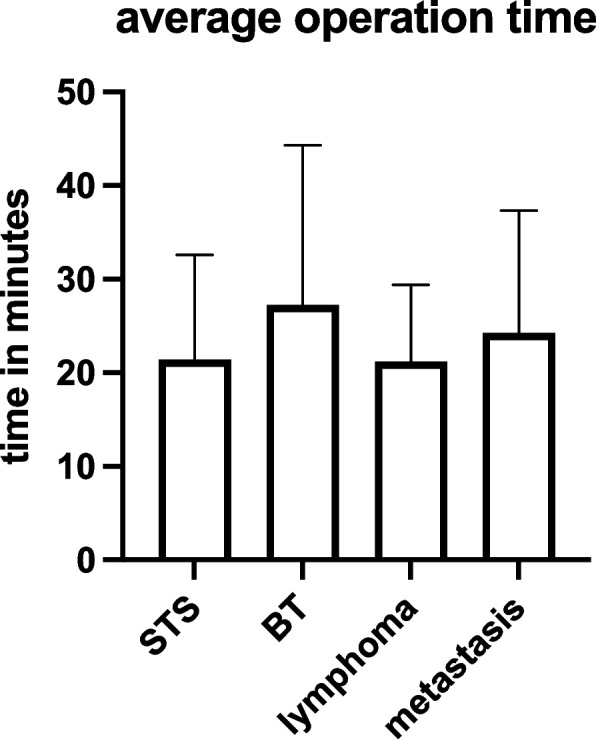
Average time of surgery in relation to the four tumor types. No significant differences were observed

Regarding the surgery´s duration, we followed the documented incision/suture times of the incisional biopsies. Here, the average time for detected soft tissue sarcomas was 21.4 (±11.2) min. The average cut/suture time of the bone sarcomas was 27.2 (±17.1) min. For lymphomas, it took on average 21.2 (±8.2) min from incision to suture. Finally, the average surgery-only time for proven metastases was 24.3 min with a standard deviation of 13 min (Fig. 6).

All incisional biopsies were performed under general anesthesia without complications. The mean time between the incisional biopsy and the final histopathological finding was 10.6 days. Only 6 of the 332 biopsies needed to be repeated because the pathohistological analysis could not produce a valid result. In the remaining 326 tumors, the incisional biopsy yielded a valid diagnosis. In 142 cases, a primary oncological resection followed. In 70 cases, the patients received adjuvant radiotherapy or chemotherapy. Mainly, because of progressive metastatic cancerous disease. All therapeutic decisions for malignant tumors were made by the interdisciplinary tumor board and for each patient individually.

The primary tumor, which was surgically removed was histopathological analyzed and there was a 100% concordance to the histopathologic diagnosis from the biopsies. All open biopsies were found to be highly accurate with a sensitivity of 97.6%. In the 2.4% cases of a frustrated or inadequate biopsy, re-biopsy was performed with evidence of malignancy. In two cases, a complication in the sense of a hematoma requiring surgical treatment was seen postoperatively. In these cases, the hematoma was evacuated promptly.

## Discussion

All patients present with unclear masses both in the ambulant setting as well as in specialized musculoskeletal centers. After appropriate imaging and finding of a suspected diagnosis, a biopsy is often necessary for confirmation to plan the further procedure. Prompt referral for further diagnostics is particularly important if a malignant disease is the possible cause of the mass. Although many biopsy techniques have been described, there is currently no standardized procedure. For example, Errani et al. described the current concepts in the biopsy of musculoskeletal tumors, which primarily include fine needle aspiration, core needle biopsy, or incisional biopsy, and came to the conclusion that there is still no clarity about the optimal biopsy acquisition [9].

Due to the low risk of complications and the lower costs, they recommended CNB to be preferred, but if this remains without a result, an incisional biopsy should be performed [9].

Once more, this shows the discordance about the validation of the different methods, especially CNB versus incisional biopsy. While some centers prefer CNB as the method of choice and publish good results in the available literature, primarily IB is used in our center with also very good results. The division of the histopathological findings into the individual tumor types makes it clear that IB shows the same sensitivity not only in soft tissue, but also in bone tissue, and has a high diagnostic confidence in solid tumors as well as in the malignancies of the hematopoietic system and metastases. From our point of view, what is important is the uncomplicated acquisition of a biopsy without a high risk of complications, the rapid availability, and the correct diagnosis. The purpose of this study was to investigate the accuracy of incisional biopsy in cases of existing suspicion of a malignant tumor of the musculoskeletal system. Especially in the case of malignant tumors, the most precise, timely, and complication-free biopsy possible is of elementary importance for the correct diagnosis and appropriate planning of further therapy [21].

Recently, Birgin et al. reported in a systematic review about diagnosing the correct soft-tissue-tumor type. The conclusion of their meta-analysis demonstrated that CNB is not inferior to IB. Sensitivity was calculated as 97% in the CNB group and 96 in the IB group for malignancy. For soft-tissue-tumors: CNB 88% and IB 93%. It is debatable whether CNB may provide higher diagnostic confidence in bone sarcoma due to its fixed position in solid tissue, whereas in soft tissue sarcomas, the displaceability of the mass, patient positioning, or the biopsy itself may significantly reduce sensitivity. In this case, open biopsy is clearly superior, as it is possible for the experienced surgeon to assess the local findings intraoperatively and still isolate and biopsy the possibly displaced soft tissue sarcoma. In conclusion and with focus on a simple technique, a high diagnostic accuracy while having less complications the meta-analysis found CNB to be the superior method [5].

In this study, all biopsies were performed in general anesthesia and by means of a surgically incisional biopsy. The incisional biopsies were highly accurate with a sensitivity of 97.6% for malignancy. In the 2.4% cases, in which a reliable histopathological diagnosis could not be made, re-biopsy was performed with evidence of malignancy. Compared to the results in literature, this can be rated as at least equal, if not even superior [5], Piya [18, 13].

Hau et al. studied the diagnostic accuracy of computed tomography (CT)-guided biopsies and fine needle aspirates of musculoskeletal lesions. They figured 359 cases out, the overall accuracy was 71%. The accuracy for 101 fine needle aspirations was 63% and for 258 CT-guided core biopsies was 74% [11].

Other studies also described that IB can have an advantage over CNB, especially in the biopsy of soft tissue tumors. Pohling et al. described in the case of soft tissue tumors a sensitivity of 100% vs. 81.8% in the comparison of CNB vs IB [19]. Regarding to the accuracy of the respective biopsy technique in soft tissue mass diagnosis, there are correspondingly controversial results in the literature: Sina et al describe a sensitivity of 100% for surgical biopsies while CNBs only reach 79.17% [14]. Another important point in addition to the accuracy of the biopsy is the complication rate of the respective biopsy technique. Many studies recommend CNB because of the supposedly lower risk of complications [13, 5, 17].

Surgery-specific risks are hematoma and surgical site infection and in relation to CNB — more pain. One also needs to consider the need for re-biopsy in the case of incorrect or unevaluable histopathology as a relevant complication and rated it as such in our study. Many authors recommend CNB over IB precisely because of the supposedly lower complication rate as well as the lower costs [5, 13, 9].

In all IB performed in this study, 8 complications occurred, of which 2 were secondary bleeding that required another operation and 6 were re-biopsies because there was no histopathological valid result. In the analyzed collectivity at the UCC was a very low risk of complications of 2.4%. The number of necessary re-biopsies with 0.6% is to be highlighted here, since from our point of view these must also be evaluated as a complication. Klein et al. showed in their study that the number of necessary re-biopsies was significantly higher in the case of CNB compared to IB (50 vs. 5; *p* = 0.003) [15].

The need of re-biopsy must be taken to account, especially in the case of malignant tumors since these delays further therapy. Also, in the Adams et al study, 6% of the diagnostic biopsies yielded no result (= re-biopsy necessary). Even more critical for patient’s welfare was a 3% rate of false diagnosis. In these cases, the biopsy described a benign tumor, which turned out to be a malignant tumor after complete resection. Patient´s survival of cancerous disease related directly to the speed of treatment [1].

Thus, an appointment for biopsy and consecutive discussion of the patient in the multidisciplinary tumor conference must be available in short time. [3].

A biopsy could be carried out just 8,8 days after the first presentation in the UCC and the final histopathological result was available after a further 10.6 days. Accordingly, the diagnosis was already available 19.4 days after the first presentation. The biopsies of this study obtained by IB further corresponded histopathological in 100% with the histopathology of the resected material, correspondingly there was a sensitivity of 100% for malignancy and a specificity of 97.6% for the tumor type. This also shows how accurate and correctly performed IBs are.

Although there is not much detailed data for comparison in the current literature, we see this as a respectable time course that enables patients to be referred quickly to the appropriate individual further treatment.

Aside from the already described necessity for re-biopsies, there were 2 postoperative bleedings in our collective that required another surgical treatment. Interestingly, these cases were not associated with a significantly longer operation time in comparison: 15 min and 24 min. All biopsies showed an average operation time of 23.5 min. There were no further complications with the general anesthesia in any case. The extended operation time of the complication courses can be best explained by a more invasive surgical procedure because of a more complex location of the tumor.

As already described, due to the extremely large heterogeneity of possible bone and soft tissue tumors of the musculoskeletal system, the type of biopsy is possible in different ways and, as already discussed leads to different results especially regarding the precision of a CNB compared to an IB. Accordingly, there are different recommendations in the literature. In the analysis of patient age performed, the validity of the data is evident. On average, the malignant bone tumors are more than 20 years younger than the patients in whom a metastasis of malignant primaries was secured. This statement agrees with the literature due to osteosarcomas and Ewing’s sarcomas occurring most frequently in young adulthood [12]. The frequency of metastases especially of gender-specific osseous metastasis is more evident in the advanced age of the patients. Interestingly, the sensitivity of our IB is independent of tumor location. Comparable to the literature, soft tissue sarcomas were clearly more frequent in the lower extremities, which can be explained by the greater amount of muscle and connective tissue.

Ultimately, an individual indication for each patient must be carefully examined. In the case of easily accessible tumors, the incision biopsy is usually a harmless method to perform with low complication and high accuracy. In the case of deeper-lying masses that may require complex and more invasive access due to their location, with corresponding soft tissue damage to be expected the possibility of a minimal invasive CNB should be examined. In case of increased risk of postoperative bleeding, CNB can be advantageous.

The overall aim should be to guarantee the respective patient the fastest and safest possible diagnostic method and thus promptly supply a definitive therapy, after a thorough discussion of the patient in a multidisciplinary tumor board.

All limitations of retrospective studies apply to this analysis. A heterogenous group of surgeons performed the IBs under supervision of an experienced musculoskeletal surgeon. The malignant specimen was resected only by a specialist with more than 5 years of experience in the field of musculoskeletal tumor surgery.

## Conclusion

Not least due to this work, the different biopsy methods are controversially discussed regarding their advantages and disadvantages. We recommend IB due to the described highest sensitivity, while in summary CNB and other more minimally invasive methods certainly have advantages in surgical effort and lower risk of complications.

Ultimately, an individual indication must be carefully considered for each patient. In particular, the surgical access route, as well as the patient’s comorbidity, seems to play a role here. For easily accessible tumors, IB is usually a harmless method with low complication and high accuracy. For deeper masses, which may require a complicated and more invasive approach due to their location, with corresponding soft tissue damage expected, the option of minimally invasive CNB should be explored. If there is an increased risk of postoperative bleeding, CNB may be beneficial.

In any case, the goal should be to provide the respective patient with the fastest and most reliable diagnosis possible and thus deliver a definitive therapy in a timely manner. For this reason, guidelines for confirming the diagnosis of soft tissue sarcomas, bone sarcomas, lymphomas, and musculoskeletal metastases should continue to include IB as the gold standard.

## Data Availability

Data can be provided upon reasonable request from the corresponding author.

## Abbreviations

BS: Bone sarcoma
CNB: Core needle biopsy
IB: Incisional biopsy
SD: Standard deviation
STS: Soft tissue sarcoma
UCC: University cancer center
